# Vitamin D and Folate as Predictors of MMSE in Alzheimer’s Disease: A Machine Learning Analysis

**DOI:** 10.3390/diagnostics11060940

**Published:** 2021-05-24

**Authors:** Giuseppe Murdaca, Sara Banchero, Alessandro Tonacci, Alessio Nencioni, Fiammetta Monacelli, Sebastiano Gangemi

**Affiliations:** 1 Department of Internal Medicine, University of Genoa, 16132 Genoa, Italy; giuseppe.murdaca@unige.it (G.M.); sara.banchero@unige.it (S.B.); alessio.Nencioni@unige.it (A.N.); fiammetta.monacelli@unige.it (F.M.); 2 Ospedale Policlinico San Martino IRCCS, 16132 Genoa, Italy; 3 Clinical Physiology Institute, National Research Council of Italy (IFC-CNR), 56124 Pisa, Italy; 4 School and Operative Unit of Allergy and Clinical Immunology, Department of Clinical and Experimental Medicine, University of Messina, 98125 Messina, Italy; gangemis@unime.it

**Keywords:** Alzheimer’s disease, biomarkers, folate, machine learning, Vitamin D

## Abstract

Vitamin D (VD) and micronutrients, including folic acid, are able to modulate both the innate and the adaptive immune responses. Low VD and folic acid levels appear to promote cognitive decline as in Alzheimer’s disease (AD). A machine learning approach was applied to analyze the impact of various compounds, drawn from the blood of AD patients, including VD and folic acid levels, on the Mini-Mental State Exam (MMSE) in a cohort of 108 patients with AD. The first analysis was aimed at predicting the MMSE at recruitment, whereas a second investigation sought to predict the MMSE after a 4 year follow-up. The simultaneous presence of low levels of VD and folic acid allow to predict MMSE, suggestive of poorer cognitive function. Such results suggest that the low levels of VD and folic acid could be associated with more severe cases of cognitive impairment in AD. It could be hypothesized that simultaneous supplementation of VD and folic acid could slow down the progression of cerebral degeneration at least in a subset of AD individuals.

## 1. Introduction

Vitamin D (VD) is a secosteroid hormone with two forms, D2 (ergo-calciferol) and D3 (cholecalciferol). The active form, 1,25-hydroxyvitamin D, also known as calcitriol, primarily regulates the homeostasis of calcium and phosphate [1,2]. According to the scientific literature, it is now well established that VD, through VD receptor (VDR) and micronutrients, including folic acid (FA), are able to modulate both the innate and the adaptive immune responses [1,2,3]. Low VD and FA levels appear to promote cognitive decline in several neurodegenerative conditions, as in Alzheimer’s disease (AD) [4,5]. Indeed, low levels of VD were found in several neurological and neuropsychiatric conditions, including those whose onset occurs during childhood, including Autism Spectrum Disorders (ASD) [6]. Low VD levels also occur in typical disorders of the older age, such as neurodegenerative processes [4]. On the other hand, VD supplementation is eventually associated with a significant cognitive function improvement in early dementias, as occurs in the case of the Mild Cognitive Impairment (MCI) [7]. Indeed, the role of VD at brain level is manifold. It is well known that VD is somewhat involved in the processes dealing with synaptic plasticity, notably in long-term potentiation, pivotal in storing information at brain level. Overall, synaptic plasticity refers to the ability to generate new synapses, eliminate existing ones, and alter the electrophysiological, molecular, and structural properties of existing synapses in response to experience [8]. As such, synaptic plasticity is key to learning and memory among other cognitive processes [9].

The mechanisms for which VD deficiency is linked to cognitive detriment are not yet fully understood. However, it was seen that a prenatal lack of VD is capable of altering genes involved in synaptic plasticity, including drebrin and neuromodulin [10,11], in turn playing a role in the pathophysiology of schizophrenia and associated cognitive impairment. On the other hand, supplementation of VD was seen to upregulate genes pivotal to synaptic plasticity. Those include synaptojanin 1, synaptotagmin 2 and calcium/calmodulin-dependent protein kinase IIδ (CaMKIIδ), beyond reporting the same action on receptors for neurotransmitters, such as dopamine, glutamate and serotonin, key for usual synaptic functioning [12].

Beyond that, VD signaling is also associated to the expression of L-Voltage-gated calcium channels (L-VGCCs), in turn acting in neurotransmitter release, changes in neuronal excitability, learning, memory, and other important physiological functions [13]. These somewhat influence long-term cognitive functions, through the modification of neuronal connectivity [14]. In addition, L-VGCCs regulate nitric oxide (NO) levels, in turn, implicated in synaptic plasticity, transmission and neuroprotection [15,16]. However, NO production is also influenced by VD levels, further boosting the importance of VD for cognitive processes, mainly learning and memory.

Aside from the effects VD has on the brain of living beings, folates are also interesting compounds when it comes to dealing with neurocognitive processes. However, their contribution in terms of cognitive improvement/preservation or, conversely, cognitive detriment, is quite debated. In fact, some works have proven a clear association between folate levels and cognitive decline (i.e., see [17]), whereas other articles have demonstrated an improvement in cognitive function in those subjects under folate supplementation, especially when displaying higher levels of homocysteine [18]. This probably occurs since concentrations of homocysteine appear to be associated with an increased risk for dementia in older adults. Conversely, the elimination of homocysteine from the body occurs thanks to two different pathways, one requiring folate and Vitamin B12, and the other one Vitamin B6 [19].

To this extent, in the present work, we analyzed a cohort of patients with AD to assess whether the levels of several blood biomarkers, including complete blood count, thyroid stimulating hormone (TSH), parathyroid hormone (PTH), vitamin B12, VD and folate can be related to functional tests and can, therefore, represent predictors of the development of AD. The analysis was performed taking advantage of the Machine Learning (ML) approach, already demonstrated to be a useful alternative to classical statistical analysis also in AD and similar conditions in the presence of high amounts of data [20]. Finally, we briefly theorized the link between VD, FA, microbiome and immune system in the etiopathogenesis of AD.

## 2. Materials and Methods

One hundred and eighteen (30 men and 88 women, median age 86 ± 5 years) AD patients were recruited from 2013 to 2020 (2013 to 2015 for the enrollment, 2016 to 2020 for the follow-up). They also signed an informed consent document proposed by the San Martino Polyclinic Hospital (which was kept in their medical records) on participation in the study. The patients were followed up at the Alzheimer Evaluation Unit outpatient clinic of the Geriatric Clinic of the San Martino polyclinic in Genoa. AD was confirmed through neuro-imaging. Inclusion criteria for the study were: presence of neurodegeneration, confirmed through TAC or brain magnetic resonance imaging (MRI), and at least two visits at the Geriatric Clinic of the San Martino polyclinic in Genoa 6 months one to another prior to the enrollment. Exclusion criteria for the study were age under 65 years old.

**Figure 1:**
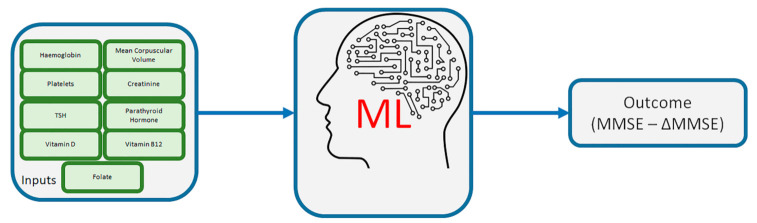
Overall view of the inputs and outcome of the ML algorithms.

To analyze the neuro-cognitive functions of the patients examined, we used the Mini-Mental State Exam (MMSE), a 30-question assessment of cognitive functioning that evaluates attention, orientation, memory, registration, recall, calculation, language and ability to draw a complex polygon [21,22]. The test consists of 11 items divided into 5 sections, its total score ranging from 0 to 30. The threshold score for “normality” is set at 24/30; however, this limit is influenced by age and education, for which correction factors have been developed [21,22]. The present study took into consideration the MMSE calculated at the first visit (MMSE1) and at the last visit (MMSE2) performed up to the year 2020. The average elapsed time between the first and the last test is 1427 days (equal to 3.9 years). A Machine Learning (ML) approach was employed to assess which of the parameters (haemoglobin, Mean Corpuscular Volume, platelets, creatinine, TSH, parathyroid hormone (PTH), vitamin B12, VD and FA) drawn from the patients were most predictive of their cognitive involvement concerned with AD (Figure 1).

To this extent, as mentioned above, the ML outcome evaluated was the MMSE, both at recruitment and after follow-up. As MMSE was taken as a continuous variable, the task demanded to the ML model was a regression task, with the evaluation of the Root Mean Square Error (RMSE) as the metrics for comparing models’ performances. According to that, the ML models, making use of 90% of data for training and 10% for test purposes, were evaluated on 10-fold cross-validation, and the best results for each model were selected as the one with the optimal trade-off between performances (in terms of minimal RMSE for the regression task) and complexity (in terms of lower number of features included in the model). This would have ensured enough generalizability to further unknown data. To do so, parameters were selected as to not simply having the minimum RMSE in absolute terms, but to have a maximum deviation of one standard error from the minimal RMSE, thus reducing complexity of the model and avoiding overfitting. The whole ML analysis was carried out under the open-source R language, using the software RStudio, version 1.3.1093 for Windows, available with the GNU Affero General Public License. Five supervised ML models (LASSO, RIDGE, Elastic Net, Classification and Regression Trees, and Random Forest) were implemented and trained, using the R-based caret package [23], allowing an unbiased comparison of regression performances between them. The models are briefly outlined below.

### 2.1. LASSO

The Least Absolute Shrinkage and Selection Operator, namely LASSO, is a very common ML model, relying on a regression analysis method. It carries out both variable selection and regularization, and aims at improving the prediction accuracy and the resulting model interpretability [24]. It is known to be particularly useful when datasets are composed of several variables hypothesized not being useful for prediction purposes.

### 2.2. RIDGE

Ridge Regression is a ML technique often employed when the regression data to be analyzed are significantly affected by multicollinearity problems. If multicollinearity occurs, it turns out that least squares estimates are totally unbiased, with a large variance, deviating them significantly from their true value. By adding a quota of bias to the regression estimates, ridge regression is able to reduce the standard errors. Conversely to LASSO, which is quite similar in some instances, RIDGE regression shrinks all the coefficients to a non-zero value.

### 2.3. Elastic Net

The Elastic Net attempts at taking the advantages of both LASSO and RIDGE, blending their optimal characteristics. Its main regularization parameter, named α, can be continuously varied between 0 and 1, with the lower limit (being zero) making the model equal to RIDGE and the upper limit (being one) to LASSO. A 0.5 value indicates a 50/50 blend between the two regression models.

### 2.4. CART

Classification and Regression Trees (CART) are popular and powerful ML models, relying on the deconstruction of the overall sample into smaller groups, performed through repeated, binary splits of the sample, considering one exploratory variable at a time.

Their advantages are manifold: they can be easily adapted to different data, including cross sectional, longitudinal, survival data, the possibility to use different types of response variables, and the fact that they do not need to make any assumptions in terms of the normality of the data distribution. On the other hand, their main limitations include their strong sensitivity to data changes and their somewhat limited interpretability.

### 2.5. Random Forest

Random Forest (RF) are learning methods that can be applied for classification and regression purposes, operating by building up a series (forest) of decision trees at the training. Their output is represented by the class that is the mode of the classes, for classification, or the mean prediction, for regression, of the individual trees [25].

With respect to the classical decision trees, RF carry on several advantages. Those include the performance of implicit on-the-run feature selection, the provision of accurate indicators of feature importance, the absence of need for particular data preparation prior to the application of the ML model, the opportunity for them to handle binary, categorical, numerical features without any need for scaling, normalization or standardization. They are also unlikely to perform overfitting, they are relatively quick to train and versatile, although their interpretability is often cumbersome.

## 3. Results

Statistical data on the parameters extracted from the patients (MMSE, blood parameters) are presented in Table 1.

The first analysis based on ML was aimed at predicting the MMSE at recruitment. According to the minimum RMSE calculated on the test set, the RIDGE model was selected as having the best regression performances, with a RMSE = 5.109. The model, whose hyperparameter lambda was optimally set at 0.15, displayed the best performances when using two input parameters (Vitamin D and folate) as the most predictive ones. The algorithm used little PC memory for the training and regression task (0.269 MB), although completing the full cycle in a relatively long amount of time (549.57 s) (Table 2). Therefore, the model is not particularly suitable in cases when a very fast response is needed to be achieved.

At follow-up, overall performances of the ML models used slightly worsened, as expected due to the higher complexity of the task caused by the amount of time elapsed between the two MMSE evaluation points. Despite its complexity and high computational cost (870.32 s elapsed time to complete the full cycle, with 3.45 MB of PC memory used), the Random Forest, using 500 trees for the forest set-up, outperformed the other models. It displayed a RMSE = 5.834 and made best use of three input parameters, being Mean Cell Volume (MCV), VD and Platelets, selected based on their predictive value. With good performances, as above, the biggest drawback of the algorithm is represented by the high computational cost, possibly decreased when selecting a lower number of trees to carry out the task demanded. To this extent, according to the simulation performed, a Random Forest composed of 150–200 trees would guarantee similar RMSE performances in a relatively shorter amount of time. The comparison between classifiers over the second task is shown in Table 3.

## 4. Discussion

According to the ML models trained and evaluated in the present work, VD appears as the most predictive with respect to cognitive impairment and, in some ways, cognitive decline, among the blood biomarkers taken into account in the analysis. Therefore, VD appears to act as a risk factor for cognitive impairment when present in the patient’s blood in low concentrations.

**Figure 2:**
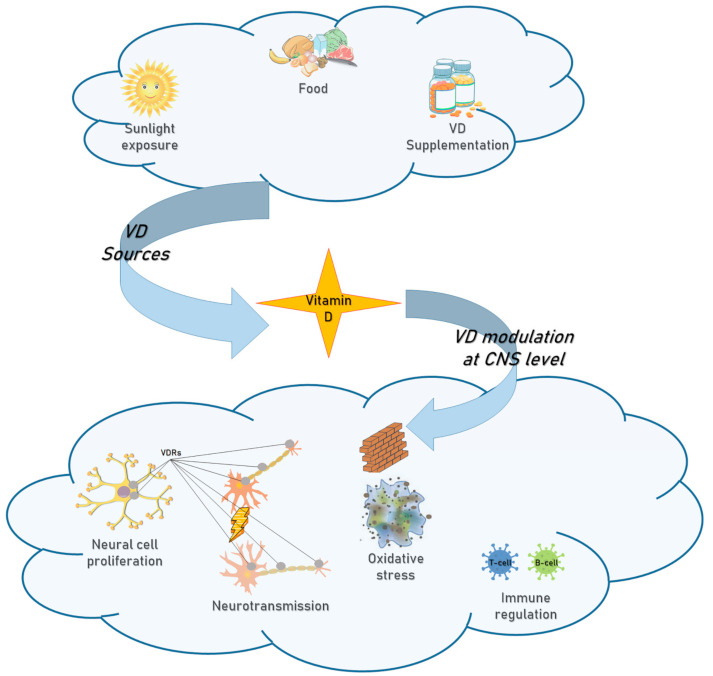
VD sources and VD modulation at CNS level.

VD regulates the adaptive immune system by inhibiting both differentiation of T lymphocytes into T-helper (Th)1 and Th17, which have a pro-inflammatory action, and of B lymphocytes into memory cells and plasma cells [26,27]. Furthermore, VD protects lymphocytes from oxidative death. Confirming this, lymphocytes from patients with very early AD and low VD levels are susceptible to H2O2-induced oxidative death [28], even before deposition of β-amyloid (Aβ) [29]. However, while in patients with mild cognitive impairment, the supplementation of VD allows, already after 6 months, to improve both lymphocyte susceptibility to death and the Aβ1-40 plasma levels, in patients with very early AD there are no benefits from VD supplementation. At the same time, also the cognitive levels of MCI individuals improved together with such supplementation, unlike observed in early AD patients. This possibly suggests efficacy of VD for the improvement of biological and cognitive status of individuals just when applied before the neurodegenerative disease onset. This result is probably due to a more advanced stage of the neurodegenerative disease or because of the intrinsic characteristics of the neurodegenerative process [28]. In addition, VD plays a role in maintaining brain integrity through phagocytosis, clearance of Aβ and decreasing of glutamate-induced neurotoxicity [12,30]. The neuroprotective action of VD is favored by the presence of VDR in neurons and glial cells [4,31]. An overall view of the action of VD at the central nervous system level is displayed in Figure 2. It has been proposed that neuronal damage from multiple insults including dyslipidemia, vascular insults, head trauma, oxidative stress, iron overload, FA deficiency could represent the primary trigger of AD. It is also supposed to induce activation of innate immune system and consequent activation of microglia and generation of pro-inflammatory cytokines (IL-1β, IFN-γ, TNF-α) [32,33]. VD, FA and gut microbiome cooperate in neuro-immune modulation. Gut microbiome synthesizes serotonin, dopamine, γ-aminobutyric acid (GABA), acetylcholine by enhancing their bioavailability in the brain [32]. On the other hand, gut bacteria species can produce amyloid peptides and lipopolysaccharides (LPS) endotoxins, capable of influencing inflammation in AD [32]. Microbiota dysbiosis increase intestinal permeability by putting the microbiota in contact with the submucosal lymphoid tissue, promoting neuroinflammation that can, in turn, lead to neurodegeneration [32]. The simultaneous presence of FA deficiency supports the activation of innate immunity and the inflammatory cascade that determines the onset of AD [34]. Notably, an association between homocysteine metabolism, oxidative stress and immune activation has been proven [34]. Low folate levels were also found to be associated with lower baseline MMSE scores in previous research [35,36].

In the present work, we have shown that the presence of low levels of VD, and in some instances the simultaneous presence of low levels of VD and FA, allow to predict MMSE, thus they are suggestive of poorer cognitive function. This is particularly true considering the MMSE scored at the time of the first assessment, that is, also the time when blood biomarkers were drawn. The predictability of blood biomarkers considering the 4 year follow-up was obviously decreased with respect to that considering the basal assessment. In addition, the usefulness of FA among the biomarkers for 4 year prediction decreased, whereas VD, this time together with MCV and platelets, remains predictive even in this complex task.

Indeed, as mentioned before, low VD levels promote episodic verbal memory, poorer reaction time/attention processing speed, focused attention/concentration and greater attention fluctuation [37,38,39]. In conclusion, according to our research, it is conceivable that levels of VD mainly, but also FA, close to the lower limits can be deleterious for cognitive functions. Thus, it is likely that the simultaneous supplementation of VD and eventually FA could slow down the progression of cognitive impairment within AD [39,40], especially when supplied early during the disease cycle.

The results of the present study should also consider a major limitation. Notably, due to the paucity of male individuals for a ML purpose, a pooled analysis for all subjects, regardless of sex/gender, was performed. In the future, with larger datasets, independent analysis for males and females can be carried out, to retrieve hidden correlations for either male or female individuals.

## 5. Conclusions and Future Directions

The present study suggests that VD and folate are possible good short- and long-term predictors for cognitive decline in patients with AD. Under such premise, it could be hypothesized that a supplementation of such compounds could help in blocking or delaying the disease progression, at least at an early stage. Related investigations could apply supplementation protocols to assess the effective response of such individuals to properly tailored treatments.

Furthermore, taking advantage of the potentialities of ML, future studies are required to increase the number of biomarkers to be evaluated in terms of predictability for the cognitive decline. This would possibly include a “multi-omics” approach to be analyzed, as well as to apply methodologies other than blood biomarkers that could be eventually easy to achieve, in a fast, non-obtrusive and economically viable fashion. Those might include brain imaging, physiological signal measurements and sensory patterns, particularly respective to those senses already demonstrated to have a significant link with neurological disorders and neurodegeneration, like smell and taste [41,42,43]. This kind of analysis could be carried out not only in patients with AD or other similar conditions, already featuring a clinically relevant stage, but also in subjects affected by MCI or Subjective Cognitive Impairment (SCI). The latter could be eventually advised early in terms of potential risk factors for neurodegeneration. This might allow their treatment with VD and/or FA supplementation in order to block or slow down the disease progression at an early stage, as reported above. The results on such groups could then be compared with those on AD patients to assess for eventual differences or similarities. This could ultimately increase the treatment effectiveness and lead to a significant benefit in terms of the improvement of the quality of life of the individuals and their family and caregivers, but also in terms of economic impact on the national health systems.

## Figures and Tables

**Table 1:** Values of the parameters extracted from the AD patients.

Parameter (u.m.)	Value (Mean ± SD)	Range (Min–Max)
MMSE, or MMSE 1	18.5 ± 6.2	5–30
MMSE follow-up, or MMSE 2	14.1 ± 7.2	4–28
Haemoglobin (g/dL)	12.6 ± 1.4	7.8–17.4
Mean Corpuscular Volume (fL/cell)	91.1 ± 5.1	62–102
Platelets (1000/µL)	195.0 ± 43.8	72–364
Creatinine (mg/dL)	0.98 ± 0.40	0.4–3.2
Thyroid-Stimulating Hormone (mIU/L)	2.2 ± 1.2	0.02–7.1
Parathyroid Hormone (pg/mL)	72.5 ± 41.3	14–337
Vitamin D (ng/mL)	29.3 ± 12.2	4–60
Vitamin B12 (pmol/L)	368.1 ± 110.0	67–927
Folate (nmol/L)	9.9 ± 5.0	1.5–41

**Table 2:** Performance of the trained classifiers on the first task (MMSE estimation).

Classifier	Hyper-Parameter(s)	Hyper-Parameter(s) Value(s) Range	Hyper-Parameter(s) Optimal Value(s)	Features Employed	RMSE
LASSO	fraction	0–1	0.01	VD	5.379
RIDGE	lambda	0–1	0.15	VD, folate	5.109
Elastic Net	fraction, lambda	0–1	0.05 (fraction), 0.01 (lambda)	VD	5.247
CART	cp	0–1	0.057	VD, MCV	5.294
Random Forest	mtry	1–9	4	VD, B12, MCV, PTH	5.636

B12: Vitamin B12; MCV: Mean Cell Volume; PTH: Parathyroid Hormone; VD: Vitamin D.

**Table 3:** Performance of the trained classifiers on the second task (MMSE estimation at follow-up).

Classifier	Hyper-Parameter(s)	Hyper-Parameter(s) Value(s) Range	Hyper-Parameter(s) Optimal Value(s)	Features Employed	RMSE
LASSO	fraction	0–1	0.1	Hb, MCV, VD	6.343
RIDGE	lambda	0–1	0.1	Hb, MCV, VD	6.431
Elastic Net	fraction, lambda	0–1	0.05 (fraction), 0.1 (lambda)	VD	6.379
CART	cp	0–1	0.08	MCV	6.807
Random Forest	Mtry	1–9	3	MCV, VD, Platelets	5.834

Hb: Haemoglobin; MCV: Mean Cell Volume; VD: Vitamin D.

## Data Availability

Data can be provided by the authors upon request.

## Abbreviations

Aβ	β-amyloid
AD	Alzheimer’s Disease
ASD	Autism Spectrum Disorders
CaMKIIδ	Calcium/calmodulin-dependent protein kinase IIδ
CART	Classification and Regression Trees
FA	Folic Acid
GABA	γ-aminobutyric acid
LASSO	Least Absolute Shrinkage and Selection Operator
LPS	Lipopolysaccharides
L-VGCC	L-Voltage-gated calcium channels
MCI	Mild Cognitive Impairment
MCV	Mean Cell Volume
ML	Machine Learning
MMSE	Mini-Mental State Exam
NO	Nitric Oxide
PTH	Parathyroid Hormone
RF	Random Forest
RMSE	Root Mean Square Error
SCI	Subjective Cognitive Impairment
Th	T-helper
TSH	Thyroid Stimulating Hormone
VD	Vitamin D
